# Paliperidone Palmitate Long-Acting Injection in Schizophrenic Patients: Improved of Negative Symtons and Metabolic Rates. 6 Years of follow up

**DOI:** 10.1192/j.eurpsy.2025.554

**Published:** 2025-08-26

**Authors:** E. S. Gisbert

**Affiliations:** PSYCHIATRY, HOSPITAL UNIVERSITARIO INFANTA SOFIA, SAN SEBASTIAN DE LOS REYES, Spain

## Abstract

**Introduction:**

PALIPERIDONE PALMITATE can improved the metabolic rates better than oral paliperidone. The negative symtoms and metabolic syndrome are a very frecuent phemomena with hard treatment.

**Objectives:**

Find the differences in the improving of negative symtoms and the metabolic rates in patients with change of neuroleptic (oral to paliperidone palmitate injection).

**Methods:**

MATERIAL AND METHODS: Observational longitudinal study during 72 months. We incluided all the patients with the diagnosis of schizophrenia, in which we done a change from oral neurolepic to Palmitate of Paliperidone and age of 18 years o more. There was a control group with paliperidone oral.

We used SANSS and CGI-Scale for negative symtoms and metabolic rates (weight, IMC, Glucose, Colestherol, TG and prolactin) and abdominal perimeter.

**Results:**

40 patients was included and only 4 refused. 56% are male. Average age 41.22 years. 24% has metabolic disease, 29% with axis IV disease and 30% with toxic substances Average dosis of palmate of paliperidone 105 mg/28 days.

The reason of change to injection was: No response 38% to oral neuroleptic treatment and side effects 28%. Rest patients wish.

Metabolic rates improved: Less in weight, IMC, Glucose, Colestherol, TG and prolactin and abdominal perimeter. These improved in the 6 years of study

The SANSS scale improved, All of them were stadistical significative (p<0,05) and CGI scale results improved.

**Conclusions:**

CONCLUSIONS The change from oral neuroleptic to palmitate of paliperidone improved metabolic rates and abdominal perimeter.

Also improved SANSS and CGI Scale.

The palmitate of paliperidone is useful in schizophrenic patients and with less incidence of metabolic side efects and improved of negative symptoms.

**Disclosure of Interest:**

None Declared

